# Double-Binding Botulinum Molecule with Reduced Muscle Paralysis: Evaluation in *In Vitro* and *In Vivo* Models of Migraine

**DOI:** 10.1007/s13311-020-00967-7

**Published:** 2020-11-17

**Authors:** Anna P. Andreou, Charlotte Leese, Rosaria Greco, Chiara Demartini, Eve Corrie, Deniz Simsek, Anna Zanaboni, Ksenia Koroleva, Joseph O. Lloyd, Giorgio Lambru, Ciara Doran, Oleg Gafurov, Elizabeth Seward, Rashid Giniatullin, Cristina Tassorelli, Bazbek Davletov

**Affiliations:** 1 Headache Research-Wolfson Centre for Age-Related Diseases, Institute of Psychiatry, Psychology and Neuroscience, King’s College London, London, UK; 2 Headache Centre, Guy’s and St Thomas’s NHS Foundation Trust, King’s Health Partners, London, UK; 3 Department of Biomedical Science, University of Sheffield, Sheffield, S10 2TN UK; 4 Translational Neurovascular Research Unit, IRCCS Mondino Foundation, Pavia, Italy; 5 Department of Brain and Behavioral Sciences, University of Pavia, Pavia, Italy; 6 Laboratory of Neurobiology, Kazan University, Kazan, Russia; 7 A.I. Virtanen Institute for Molecular Sciences, University of Eastern Finland, Kuopio, Finland

**Keywords:** Migraine, botulinum, trigeminal, trigeminovascular, glyceryl trinitrate model, multivalent, neuronal delivery

## Abstract

With a prevalence of 15%, migraine is the most common neurological disorder and among the most disabling diseases, taking into account years lived with disability. Current oral medications for migraine show variable effects and are frequently associated with intolerable side effects, leading to the dissatisfaction of both patients and doctors. Injectable therapeutics, which include calcitonin gene–related peptide–targeting monoclonal antibodies and botulinum neurotoxin A (BoNT/A), provide a new paradigm for treatment of chronic migraine but are effective only in approximately 50% of subjects. Here, we investigated a novel engineered botulinum molecule with markedly reduced muscle paralyzing properties which could be beneficial for the treatment of migraine. This stapled botulinum molecule with duplicated binding domain—binary toxin-AA (BiTox/AA)—cleaves synaptosomal-associated protein 25 with a similar efficacy to BoNT/A in neurons; however, the paralyzing effect of BiTox/AA was 100 times less when compared to native BoNT/A following muscle injection. The performance of BiTox/AA was evaluated in cellular and animal models of migraine. BiTox/AA inhibited electrical nerve fiber activity in rat meningeal preparations while, in the trigeminovascular model, BiTox/AA raised electrical and mechanical stimulation thresholds in Aδ- and C-fiber nociceptors. In the rat glyceryl trinitrate (GTN) model, BiTox/AA proved effective in inhibiting GTN-induced hyperalgesia in the orofacial formalin test. We conclude that the engineered botulinum molecule provides a useful prototype for designing advanced future therapeutics for an improved efficacy in the treatment of migraine.

**Supplementary Information:**

The online version contains supplementary material available at 10.1007/s13311-020-00967-7.

## Introduction

Migraine [1] is a disabling and undertreated disorder, affecting ~ 15% of the general population [2, 3], with a significant financial burden for many countries [4, 5]. Studies in animal models have uncovered several brain networks that are involved in migraine pathophysiology, including the hypothalamus [6–9], the occipital cortex [7, 10–13], and the brainstem [7, 14, 15]. Although it remains unclear which molecular events initiate migraine attacks, there is a general agreement that a successful treatment strategy will involve inhibition of the peripheral trigeminal fibers innervating pain-producing extracranial and intracranial structures.

A number of unspecific oral preventive medications are available for patients with frequent or chronic migraine [16]. However, their side effects, need for daily intake, and variable responses lead to dissatisfaction of both patients and the treating consultants. In the last decade, injections of botulinum neurotoxin A (BoNT/A) gained FDA approval for preventive treatment of chronic migraine (Botox, Allergan Inc., Irvine, CA) [17–19], i.e., migraine occurring on at least 15 days/month for more than 3 months [1]. Clinical studies demonstrated that a series of Botox injections around the scalp lead to a therapeutic improvement quantifiable as a ≥ 50% reduction of headache days in about 50% of patients with chronic migraine [20–23]. The clinical efficacy and minimally invasive injections every 3 months have made BoNT/A an important treatment option for patients with chronic migraine.

The precise molecular events that are affected by BoNT/A in reducing migraine symptoms are not fully understood; however, it is generally agreed that, in the treatment of migraine, inhibition of the peripheral trigeminal fibers is of pivotal importance. BoNT/A is known as a strong paralyzing substance, and therefore, its efficacy in the treatment of chronic migraine is hampered by the dose-limiting concern about muscle paralysis that occurs even at low nanogram doses [24]. BoNT/A cleaves its specific neuronal target—synaptosomal-associated protein 25 (SNAP-25), a member of the SNARE (soluble *N*-ethylmaleimide–sensitive factor attachment protein receptor) family of proteins, preventing the correct assembly of the SNARE complex which leads to a potent blockade of neurotransmitter and neuropeptide release. At the neuromuscular junction, BoNT/A-induced cleavage of SNAP-25 inhibits the release of acetylcholine from the nerve endings, resulting in muscle paralysis [25]. Similarly, by cleaving SNAP-25, BoNT/A can interfere with sensory neuronal secretion by blocking the presynaptic release of neuropeptides and neurotransmitters [26–28]. In animal models of migraine, BoNT/A was shown to block the release of calcitonin gene–related peptide (CGRP) and glutamate from trigeminal ganglion neurons [26–28]. In the trigeminovascular model of migraine, BoNT/A was shown to block the mechanical activation and sensitization of C fibers [27, 29], as well as the chemical activation of neurons upon stimulation of TRPV1 and TRPA1 receptors [30]. However, the highly paralyzing nature of native BoNT/A presents an obstacle to achieving a maximal therapeutic window in the treatment of chronic pain conditions.

Development of anti-nociceptive drugs that can target neurons involved in migraine and prevent this condition, with fewer side effects, remains an unmet need. To improve the efficacy and safety of BoNT/A, we introduce here a prototype synthetic BoNT/A molecule with two receptor-binding domains for strong binding to neurons. The new molecule, named binary toxin-AA (BiTox/AA), is a larger molecule than native BoNT/A, and this potentially could reduce its paralytic activity due to reduced penetration into the tight neuromuscular junctions. BiTox/AA was investigated here in cell assays and in migraine animal models. Our data show that BiTox/AA has similar efficacy in cleaving SNAP-25 as BoNT/A but acts with a markedly lower muscle paralyzing effect.

## Methods

### Animals

All experiments described here meet the European Community Council Directive of September 22, 2010 (2010/63/EEC). The corresponding ethics committees for the use of animals in research in each of the countries and universities participating in this project also approved all experimental protocols. The IASP’s guidelines for pain research in animals were followed [31]. Experiments were carried out in male Sprague-Dawley rats aged 6–10 weeks. All rats were housed on a 12 h/12 h light/dark cycle with food and water available *ad libitum*, at an ambient temperature of 22 °C.

### BiTox/AA Production and Testing in Human Neuroblastoma Cells

Preparation of BiTox/AA involved recombinant production of 3 structurally independent units: the receptor-binding domain (HcA, BoNT/A 874–1296, UniProt K4LN57) independently fused to synaptobrevin (rat syb2 25–84, UniProt P63045) or syntaxin (rat syx 3, 195–253, UniProt Q08849) peptides and the light-chain translocation domain (LHn) fused to SNAP-25 as described previously, similar to the preparation of BiTox/A [32]. These recombinant proteins were expressed in bacteria, purified, and then mixed to form BiTox/AA. Specifically, proteins were expressed in a BL21 strain of *Escherichia coli* as glutathione *S*-transferase C-terminal fusions. Proteins fused to glutathione *S*-transferase were purified on Glutathione Sepharose beads (GE Healthcare, Buckinghamshire, UK) and eluted from beads in 20 mM HEPES, pH 7.3, and 100 mM NaCl (buffer A) using thrombin. Further purification was achieved by gel filtration using a Superdex 200 10/200 GL column (GE Healthcare). The BiTox/AA was assembled by mixing the three fusion proteins for 60 min at 20 °C, each component at 1 μM concentration, in buffer A containing 0.4% octyl glucoside. After confirmation of the assembly by SDS-PAGE and Coomassie staining, the protein was aliquoted and stored at − 80 °C before functional experiments. Cleaved SNAP-25 was analyzed by Western immunoblotting of human SiMa neuroblastoma cells treated with BiTox/AA or native BoNT/A for 3 days as previously described [32].

### EMG Analysis of Rat Gastrocnemius Muscle

Eight- to ten-week-old rats were lightly anaesthetized with 2–4% isoflurane. Stimulating needle electrodes (ELSTM2; Biopac, Goleta, CA) were inserted perpendicularly into the muscle approximately 0.5 cm from the fifth lumbar vertebrae on either side. The anode was always placed distally and the cathode placed proximally to the recording leg. A ground electrode (EL452, Biopac) was placed in the base of the tail. A reference recording needle electrode (EL450, Biopac) was placed over the tendon of the gastrocnemius muscle, and a recording electrode was placed in the belly of the medial gastrocnemius muscle.

Compound muscle action potential (CMAP) measurements were performed using a Biopac system with a bandpass of 30–9999 Hz and 200× gain. A 0.2-ms pulse stimulation was performed with a voltage stimulator (BSLSTMB). Supramaximal stimulation was determined for each recording. The amplitude of the CMAP waveform was then measured. Eight recordings per leg were performed, and the largest three recordings were averaged.

Baseline CMAP recordings were determined for the gastrocnemius of each hind limb. Groups of three rats were each injected with 2 U, 6.3 U, 20 U, or 63 U BoNT/A (150 kDa, Metabiologics; 1 U = 3.7 pg) or 1.5 ng, 5 ng, 16 ng, 50 ng, or 160 ng BiTox/AA (30 μl injection volume) subcutaneously over the recording site of the left gastrocnemius muscle using a BD Micro-Fine 0.5-ml insulin syringe immediately after baseline recording. CMAPs were then recorded from both gastrocnemius muscles again on days 1, 2, 3, and 7, and the fold change in CMAP at each time point was calculated based on the baseline CMAP recording for each rat individually.

### Primary Culture of Trigeminal Neurons and Immunocytochemistry

Trigeminal ganglia were dissected from 6–8-week-old rats and digested for 1 h and 20 min at 37 °C with 1 mg/ml Dispase II (D4693, 0.85 U/mg; Sigma, St. Louis, MO) and 0.6 mg/ml collagenase XI. Enzymes were dissolved in 155 mM NaCl (S/3160/60; Fisher Scientific, Loughborough, UK), 4.8 mM HEPES sodium salt (H8651, Sigma), 5.6 mM HEPES (B299-500; Fisher BioReagents, Loughborough, UK), 1.5 mM KH_2_PO_4_ (P/5240/53, Fisher Scientific), and 10 mM d-(+)-glucose (G7528, Sigma). Digested ganglia were triturated with a P1000 pipette, and the suspension was layered on top of 4 ml of 15% bovine serum albumin (A2153, Sigma) in wash media then centrifuged at 110*g* for 5 min. Wash media contained DMEM/F-12 with GlutaMax (31331; Gibco, Loughborough, UK), 10% heat-inactivated horse serum (26050, Gibco), and 1% penicillin–streptomycin (P0781, Sigma). The supernatant was removed, and trigeminal ganglion neurons were resuspended in 5 ml of wash media and centrifuged at 110 RCF for 5 min. The supernatant was removed, and the neurons were resuspended in complete media, containing Neurobasal A medium (10888, Gibco), 1% fetal bovine serum (10500064, Gibco), 1% penicillin–streptomycin (P0781, Sigma), 20 ng/ml NGFβ (SRP4304, Sigma), 1× B27 (17504-044, Gibco), 1% GlutaMax (35050-061, Gibco), 20 μM uridine (U3003, Sigma), and 20 μM 5′-fluoro-2′-deoxyuridine (F0503, Sigma). Trigeminal neurons were plated on μClear 96-well plates (655090, Greiner, Kremsmünster, Austria) which had been coated with 10 μg/ml laminin for at least 2 h at 37 °C. Neurons were cultured for 7 days, with a half-medium change at day 3. At DIV7, wells were treated with 2 nM BiTox/AA or the vehicle control (*n* = 4 from two animals, *N* = 2).

Immunocytochemistry of trigeminal ganglion neurons was performed in 96-well plates (Greiner μClear). Trigeminal neurons were washed once with ice-cold phosphate-buffered saline then fixed with 4% paraformaldehyde for 10 min, all on ice. Neurons were washed with PBS once at 20 °C then permeabilized for 15 min with 0.1% Triton X-100 (BP151, Fisher, Loughborough, UK). Wells were washed with PBS twice, then blocking solution containing 2% fish skin gelatin (G7765, Sigma), 0.1% Tween 20 (BP337, Fisher), and 2% BSA (A2153, Sigma) in PBS was added for 1 h. Mouse anti-beta III tubulin (MAB1195; R&D Systems, Abingdon, UK) at 1:2000 dilution and rabbit anti-cleaved SNAP-25 (Davletov lab, raised against a synthesized peptide of SNAP-25 190–197 (TRIDEANQ)) at 1:5000 dilution in blocking solution were applied to the neurons for 1 h. Wells were washed twice with PBS, then Alexa Fluor 488 goat anti-mouse (A11029; Invitrogen, Carlsbad, CA) and Alexa Fluor 594 goat anti-rabbit (A11012, Invitrogen) were diluted at 1:2000 and DAPI was diluted at 1:10,000 in blocking solution and applied to the trigeminal neurons for 45 min protected from light. Wells were washed twice in PBS then imaged at × 40 with a Leica DMIRB inverted epifluorescence microscope and a Hamamatsu C4742-95 camera.

### Electrophysiology in Hemiskull Preparations

Rat hemiskull preparation (*n* = 12 hemiskulls from 12 animals) for direct recording of action potentials from trigeminal nerve ending was performed as described earlier [33, 34]. Briefly, rats were euthanized with carbon dioxide, and after decapitation skin and flesh were removed, lower jaw was dissected. Skull was cut sagittally, and brain was removed from hemiskulls, while paying maximum attention to leave meninges untouched. In the recording chamber, hemiskulls were continuously perfused (6 ml/min) with oxygenated (5% CO_2_/95% O_2_) isotonic artificial cerebrospinal fluid (aCSF) (119 mM NaCl, 30 mM NaHCO_3_, 11 mM glucose, 2.5 mM KCl, 2 mM CaCl_2_, 1 mM MgCl_2_, 1 mM NaH_2_PO_4_). Next, through a small incision in the dura mater, the nervus spinosus of the mandibular branch of the trigeminal nerve was picked up with a recording glass electrode (~ 150 μm inner diameter, resistance ~ 1 M when filled with aCSF). The same electrode was used for recording of action potentials generated in distal parts of transected nervus spinosus. Reference silver electrode was dipped into the bath with hemiskull preparation. At the beginning of each experiment, 10 min of spontaneous activity was recorded (baseline/control). BiTox/AA was applied in aCSF, and hemiskulls were superfused with isotonic aCSF for 20 min (washout). Recordings were taken with a low-noise digital amplifier (ISO 80; WPI Inc., Sarasota, FL) with the following parameters: bandpass of 300 Hz–3 kHz and gain of 10,000. All recorded signals were digitized at 125 kHz using a NIPCI 6221 data acquisition (DA) board (National Instruments, Austin, TX). WinEDR software (Strathclyde University, Glasgow, UK) was used for signal visualization during experiments. Spike clustering was performed as previously described [34]. In brief, prior to data analyses, experimental recordings were filtered by the digital Chebyshev type II filter. A 20-s-long spikeless interval at the beginning of each experiment was used for calculating the baseline noise variance for spike detection and scaling to enable averaging of the data across multiple experiments. The recording was considered to contain a spike when its amplitude was greater than 4 standard deviations of the baseline noise. Recording amplitude was normalized by baseline noise and expressed in arbitrary units. For each spike, we calculated its amplitude and temporal parameters of the positive and negative phases (rise time, decay time, spike areas, and duration) [34]. Further, spike analysis was performed using a custom-made program written in MATLAB (MathWorks, Natick, MA).

### Extracellular Single-Cell Recording from Trigeminal Ganglia

BiTox/AA (20 ng) or saline (total volume, 100 μl) was injected in a blinded fashion in the periorbital area of lightly anaesthetized rats under isoflurane (1–2%). Seven days later, rats were anaesthetized with an intraperitoneal injection of 60 mg/kg pentobarbital sodium (Merial, Harlow, UK), and general anaesthesia was maintained with continuous intravenous infusion of pentobarbital (12–15 mg/kg/h). A tracheotomy was performed to permit ventilation of the animal, and end-tidal expired CO_2_ was monitored and maintained between 3.5 and 4.5% (CapStar-100; CWE, Oxford, UK). The left femoral vein and artery were cannulated to allow for constant intravenous infusion of anaesthetic and monitoring of blood pressure, respectively. Adequate anaesthesia was judged by the absence of toe-pinch withdrawal and eye-blink reflexes and gross changes in blood pressure. Core temperature was monitored and maintained near 37 °C using a homoeothermic blanket system (TC-1000, CWE). The animal was fixed on a stereotaxic frame (Kopf Instruments, Tujunga, CA). Craniotomies were performed to expose the middle meningeal artery and to allow access to the recording electrode. Extracellular activity from single units in the trigeminal ganglia, accessed stereotaxically, was recorded using a glass-insulated tungsten microelectrode (Kation Scientific, Minneapolis, MN) with an impedance of ~ 1 MΩ. Signals were amplified and filtered as previously described [35]. The conditioned signal was digitized for storage on a computer using a Micro 1401-3 with Spike2 software (CED, London, UK). Evoked potential activation thresholds of single first-order neurons (~ 3–5 neurons per animal) were assessed following electrical stimulation of the middle meningeal artery (1–30 V), via a Grass S88 stimulator (Grass Instrumentation, West Warwick, RI) to activate trigeminovascular afferents. Evoked potentials represent an *all* or *nothing* event, allowing assessment of the threshold of action potential activation by ramping up the stimulating voltage, while keeping the pulse width constant (0.1 ms). Mechanical activation threshold was assessed using von Frey filaments of increasing force (up to 15 g) on the periorbital area over the receptive field, until an action potential was recorded on a live data recording system. All cells included in this study responded to electrical stimulation with latencies consistent with Aδ fibers (typically 7–10 ms) or C fibers (typically 15–70 ms). Recordings were made from cell bodies and were characterized by their unfiltered biphasic action potential morphology [36]. Cells received a nociceptive-specific mechanoreceptor input from cutaneous receptive fields on the face. The receptive fields were all ipsilateral and involved the ophthalmic (first) division of the trigeminal nerve.

### Orofacial Formalin Test

Eight-week-old rats (SD; Charles River, Calco, Como, Italy) weighing 235–240 g (*N* = 5–10/group) were used in behavioral testing. Rats were housed in plastic boxes in groups of 2 with water and food available *ad libitum* at the centralized animal facility of the University of Pavia, Italy. All procedures were conducted in accordance with the European Convention for Care and Use of Laboratory Animals, and the experimental protocol was approved by the Italian Ministry of Health (Document N°1032/2015-PR). BiTox/AA (10 ng) or vehicle (saline; total volume, 25 μl) was injected into the right upper lip of rats. Seven days after treatment, rats were injected with glyceryl trinitrate (GTN) or its vehicle and tested at the orofacial formalin test 4 h later. GTN (Bioindustria L.I.M., Novi Ligure, Italy) was prepared from a stock solution of 5 mg/1.5 ml dissolved in 27% alcohol and 73% propylene glycol. The GTN solution was diluted in saline (0.9% NaCl) to reach the final concentration of alcohol of about 6% and that of propylene glycol of 16% and was administered intraperitoneally (i.p.) at a dose of 10 mg/kg. The GTN vehicle (i.p.) contained saline, 6% alcohol, and 16% propylene glycol. Before behavioral test, all rats were acclimatized to the test chamber for 20–25 min. For the quantification of the nocifensive behavior, we used off-line analysis of the videos recorded by a camera located 50 cm from the observation box to offer a clear view of the rat. The subcutaneous injection of formalin (1.5%, 50 μl) was performed into the right upper lip. Immediately after formalin injection, each animal was placed into the observation box and its behavior recorded for a 45-min period [37]. Face rubbing was measured by counting the seconds the rat spent grooming the injected area with the ipsilateral forepaw or hindpaw during the first 6 min after formalin administration for phase I, and then from min 12 to min 45 for phase II. Researchers who performed the evaluations were blind to treatments.

### Statistical Analysis

For the comparison of BiTox/A and BiTox/AA in SiMa neuroblastoma cells, the differences between groups were analyzed by two-way analysis of variance (ANOVA) followed by Sidak’s multiple comparisons test. To compare the effect of BiTox/AA on basal and 4-AP–induced spiking activity in the meningeal afferents, we used unpaired *t* test. For the behavioral response following the GTN–orofacial formalin test, the differences between groups for each phase (GTN + vehicle *vs* GTN + BiTox/AA) were analyzed by unpaired *t* test. Electrical and mechanical action potential thresholds in the trigeminovascular activation model of migraine were compared between groups with non-parametric statistics using the Mann–Whitney *U* test. Differences were considered to be significant when *p* is < 0.05.

### Disclosures

The investigators have disclosed any potential conflicts to all study participants in the Acknowledgments section of this manuscript.

## Results

### Synthesis of BiTox/AA and Its Functional Evaluation

**Fig. 1:**
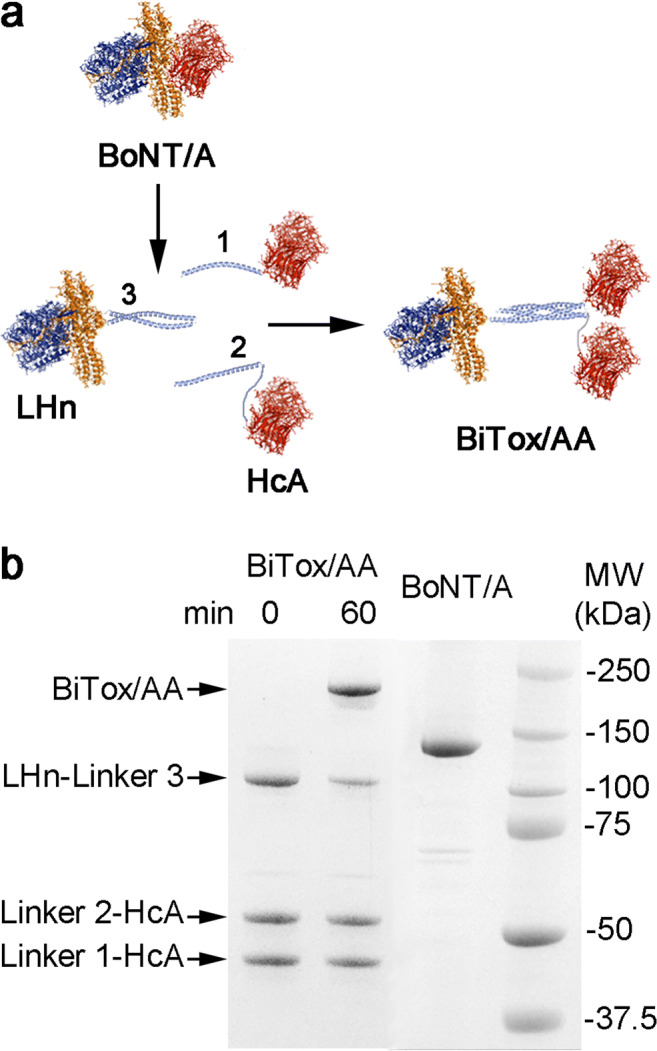
Synthesis of BiTox/AA. (**a**) Schematic showing the native BoNT/A and formation of BiTox/AA from three individual fusion proteins. LHn is the botulinum type A protease with its translocation domain, and HcA is the binding domain of BoNT/A. The three linking peptides form an irreversible complex (light blue). (**b**) Coomassie-stained SDS-PAGE gel showing the formation of BiTox/AA after the 60-min assembly reaction. The lane indicating 0 min demonstrates the initial protein amounts used in the BiTox/AA assembly reaction. BiTox/AA exhibits higher molecular weight compared to the native BoNT/A molecule. Excess amounts of HcA with linkers 1 and 2 in the lane 60 min migrate at their original positions. Molecular weight (MW) standards are shown on the right

In a recent study, we introduced a non-paralyzing BiTox/A molecule which exhibited anti-nociceptive properties in the range of 50–200 ng but was inferior to native BoNT/A in the cleavage of SNAP-25 when assessed in neuronal cultures [38]. Here, we used the same *stapling* system to prepare a novel botulinum type A molecule carrying two binding domains, unlike the previous BiTox/A. For protein linking, we used three shortened SNARE helical polypeptides called linkers 1, 2, and 3. These three linkers assemble spontaneously within 1 h into a highly stable helical bundle permitting on-demand conjugation of proteins (Fig. 1a). We fused the binding domain of BoNT/A (HcA) separately to linkers 1 and 2, while linker 3 was fused to the botulinum type A protease with its translocation domain (commonly known as LHn). All three fusion proteins were purified by affinity chromatography and gel filtration on a Superdex 200 column. Mixing the two binding domains in the presence of LHn–linker 3 led to the formation of an SDS-resistant protein, named here BiTox/AA, which exhibited higher molecular weight compared to native BoNT/A due to the presence of the linking system and the additional binding domain (Fig. 1b).

**Fig. 2:**
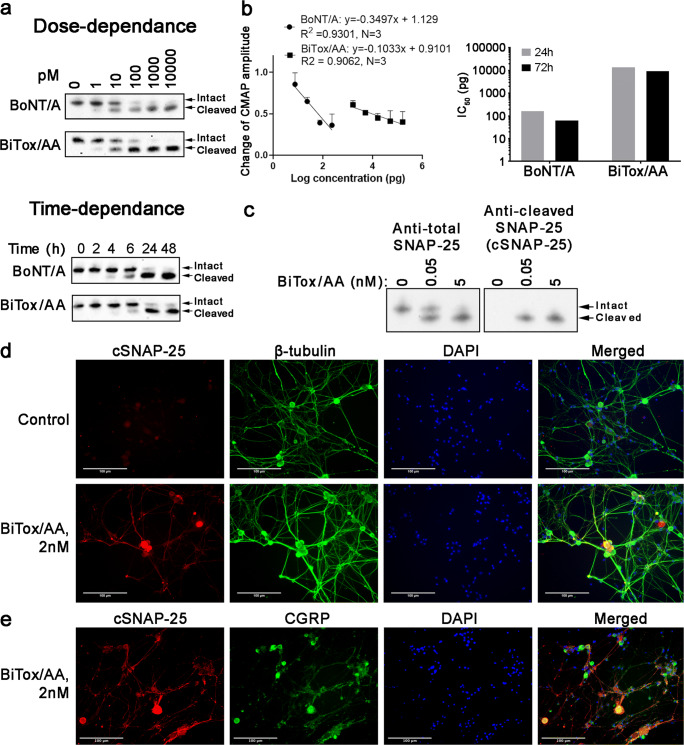
Functional evaluation of BiTox/AA in neuronal cultures. (**a**, Upper panel) example immunoblot of human SiMa neuroblastoma cells treated with BiTox/AA or BoNT/A for 72 h using an anti-SNAP-25 antibody (*n* = 3). Note the downward molecular shift of SNAP-25 upon the action of botulinum protease correlating with its increasing doses. Positions of the intact and cleaved SNAP-25 are indicated. (**a**, Lower panel) example immunoblot of SiMa neuroblastoma cells treated with either 1 nM BiTox/AA or 1 nM BoNT/A for the indicated duration of time using an anti-SNAP-25 antibody (*n* = 3). (**b**) Graph showing the change in CMAP amplitude measured by EMG in rats 72 h after injection with varying doses of BiTox/AA and BoNT/A, compared to the baseline amplitude (left panel). Bar chart showing the dose difference in logarithmic scale required to achieve 50% reduction in CMAP values measured 24 h and 72 h post injection (right panel). (**c**) Example immunoblot showing the specificity of the cSNAP-25 antibody to the cleaved end of SNAP-25, as compared to antibody raised against the whole SNAP-25 protein. (**d**) Examples of fluorescent micrographs of cultured rat trigeminal neurons treated with either vehicle or BiTox/AA and co-immunostained using the cSNAP-25 antibody and an anti-tubulin antibody. (**e**) BiTox/AA-cleaved SNAP-25 co-localizes with a subset of CGRP neurons in rat trigeminal neuron culture

Next, we compared the cleavage of neuronal SNAP-25 by BiTox/AA against native BoNT/A in human neuronal cultures derived from SiMa neuroblastoma cells. Figure 2a shows that dose dependence and the time-dependent rate of the cleavage of SNAP-25 were very similar between BiTox/AA and native BoNT/A. We previously found that BiTox/A with single binding domain exhibited a significantly compromised paralytic activity which could be due to impeded penetration into the tight neuromuscular junctions or into small synaptic vesicles operating in motor neurons. Therefore, we investigated the properties of BiTox/AA after injection into the muscle, using electromyography to determine changes in CMAP in the gastrocnemius muscle of rats 24 h and 72 h post injection. Figure 2b shows that BiTox/AA was around 100 times less paralyzing than BoNT/A, based on the dose required to reduce CMAP amplitude to 0.5 of the baseline levels (165 pg (24 h) or 62 pg (72 h) for BoNT/A, 14.1 ng (24 h), or 9.3 ng (72 h) for BiTox/AA).

Next, we investigated the action of BiTox/AA in rat trigeminal neurons in culture. At day 7 after dissection, the neurons were treated for 24 h with 2 nM BiTox/AA or control buffer and cleaved SNAP-25 was visualized using rabbit polyclonal antibody recognizing only the botulinum-cleaved end of SNAP-25 (Fig. 2c). Figure 2d shows that cleaved SNAP-25 can be detected upon the addition of BiTox/AA in some trigeminal neurons, identified by co-staining with the general neuronal marker beta-III tubulin. Importantly, we observed a co-localization of BiTox/AA-cleaved SNAP-25 with CGRP neurons (Fig. 2e). Together, we conclude that duplication of the binding parts of BoNT/A with a simultaneous increase in the molecular structure allows production of a lesser-paralyzing agent that is still able to target sensory neurons implicated in migraine biology.

### Functional Assays in *In Vitro* Migraine Models

**Fig. 3:**
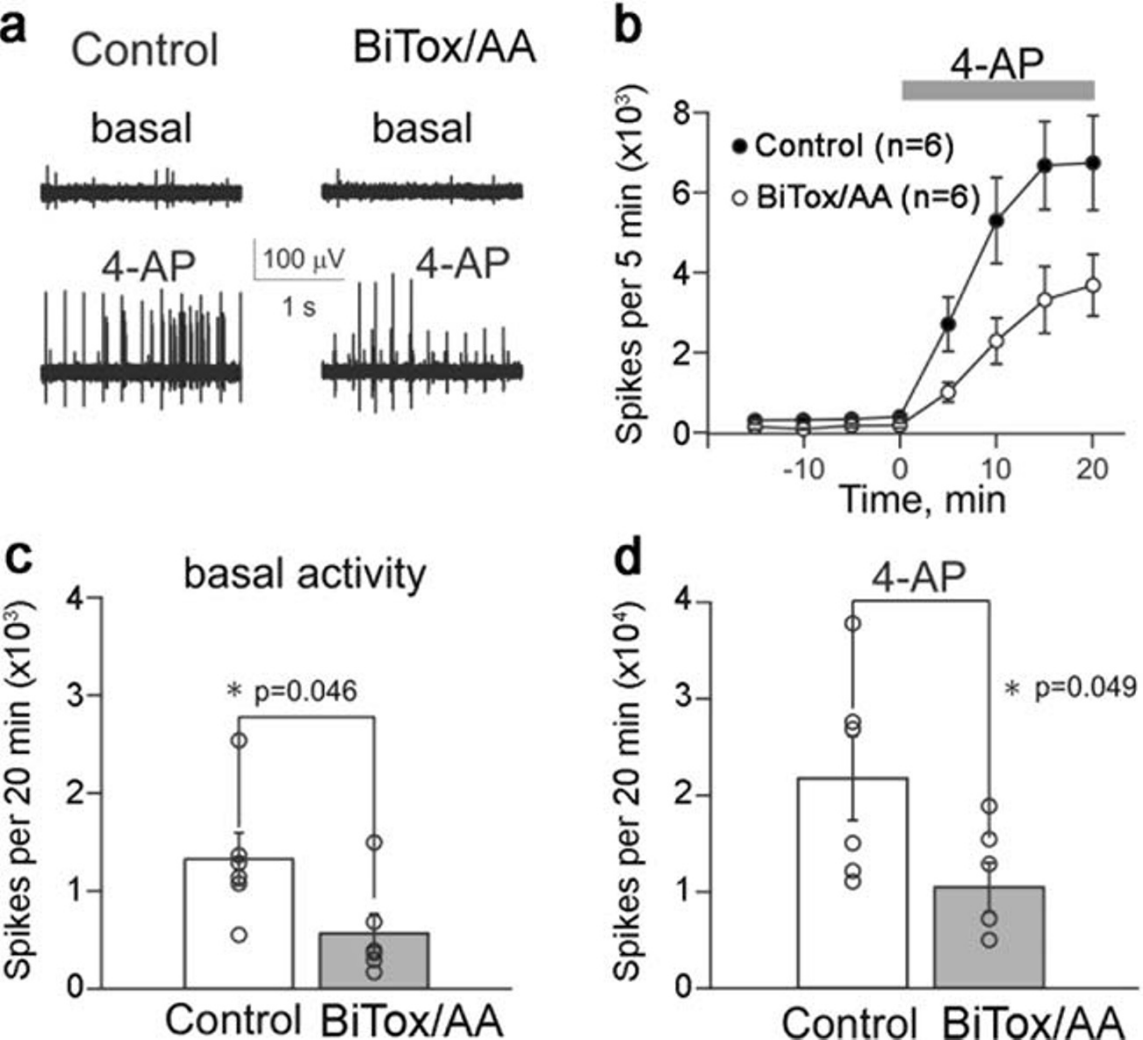
Inhibitory effect of BiTox/AA on spiking activity in rat trigeminal meningeal preparation. (**a**) Examples of basal and 4-AP–induced spiking activities in the meningeal afferents in control conditions (left) and after 6 h of exposure to BiTox/AA (right). (**b**) The time course of 1 mM 4-AP action in control condition and after 6 h of exposure to BiTox/AA (*n* = 6 for both conditions). (**c**) Bar chart showing the number of spontaneous spikes in a 20-min recording in control conditions (white) and after application of BiTox/AA (gray, mean ± S.E.M., *n* = 6, *p* = 0.046, *t* test). (**d**) Bar chart showing the number of spikes during the 20-min action of 4-AP in control condition (white) and after exposure to BiTox/AA (gray, *n* = 6, *p* = 0.049, *t* test)

Migraine pathophysiology is believed to involve activation of trigeminal fibers innervating the meninges [39]. Recordings of the firing rate of trigeminal fibers in meningeal preparations are an established method for assessing the efficacy of potential treatments in suppressing trigeminal fiber activation. The effect of BiTox/AA on nociceptive firing in meningeal afferents was evaluated in basal conditions and after activation of nerve fibers with 4-AP. The latter was selected to robustly unmask the activity in most of the fibers of the meningeal nerve through an inhibitory action of 4-AP on voltage-gated A-type and delayed-rectifier (DR) potassium channels (K_(A)_ and K_(DR)_) abundantly expressed in sensory neurons. Figure 3a shows electrical activity of nerve fibers in hemiskull preparations of rats in the absence and presence of 4-AP and before (left) and after (right) exposure for 6 h to BiTox/AA at a concentration of 10 nM. After application of 1 mM 4-AP to an innervated area of meninges, the spiking activity was dramatically increased, eventually forming the very regular *pulse-like* patterns of activity (Fig. 3a). The inhibitory effect of BiTox/AA on 4-AP–activated nerve fiber activity was significant for all time points as shown in Fig. 3b. Figure 3c shows that the basal activity was 1357 ± 276 spikes during 20 min of observation (*n* = 6), whereas after BiTox/AA treatment, the basal activity of nerve spikes was reduced to 579 ± 203 (*n* = 6, *p* = 0.046). After application of 4-AP, the number of spikes in control experiments increased to 21,934 ± 4401 during a 20-min interval (*n* = 6, Fig. 3d). In BiTox/AA-pretreated hemiskull preparations, the increase of spiking activity after 4-AP was halved (10,530 ± 2560 spikes during 20 min, *n* = 6, *p* = 0.049, Fig. 3d). In summary, these results indicate that BiTox/AA reduces both spontaneous and evoked activities in meningeal sensory afferents, previously implicated as the site of migraine pain generation.

### Trigeminovascular Model of Migraine

**Fig. 4:**
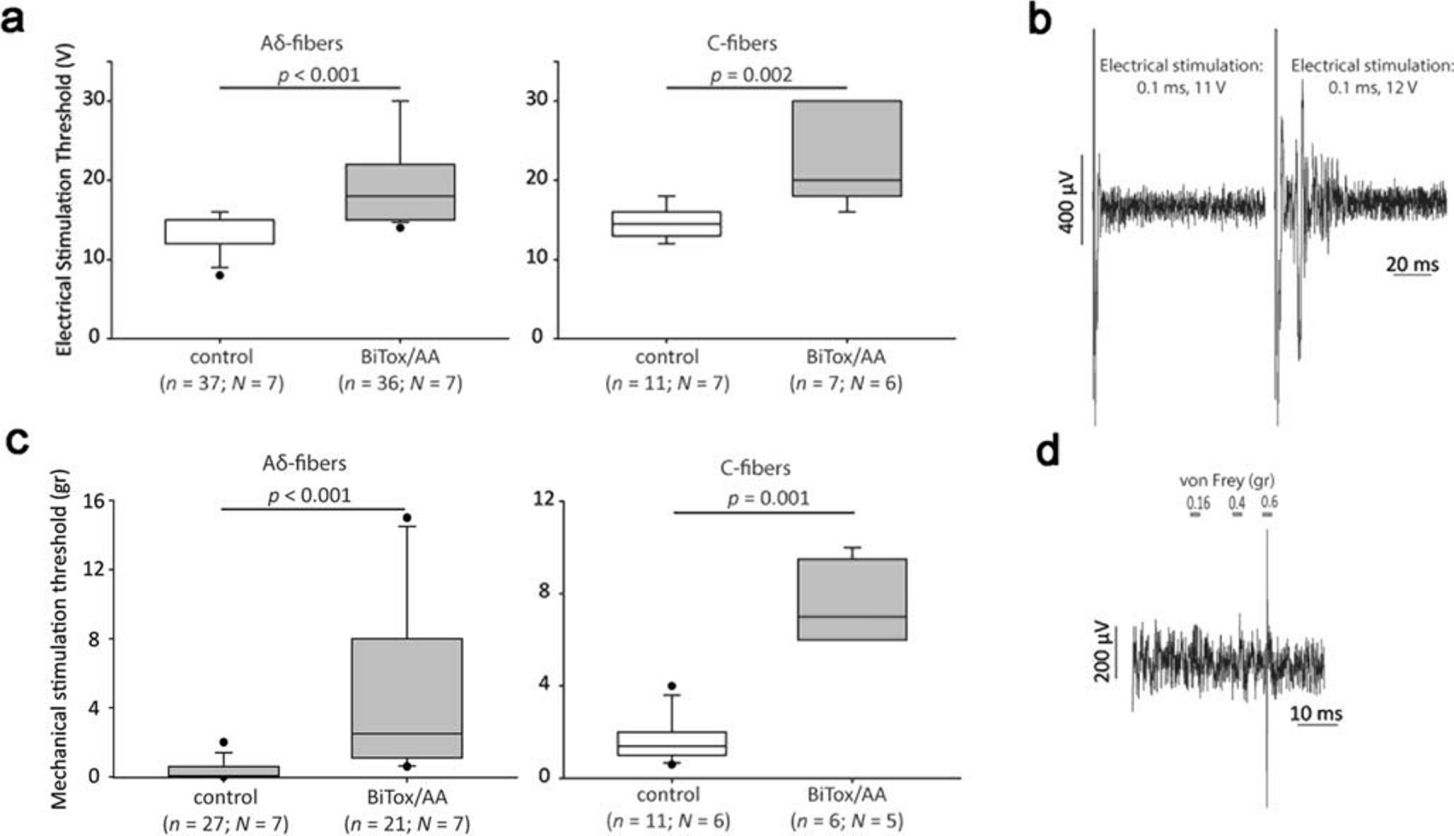
Analysis of the inhibitory effect of BiTox/AA on electrical and mechanical activation thresholds of rat primary trigeminal neurons in the trigeminovascular model of migraine. (**a**) Treatment with BiTox/AA significantly increased the electrical stimulation threshold required to induce an action potential recorded *in vivo* from trigeminal neurons with Aδ-fiber (*p* < 0.001, Mann–Whitney *U* test) and C-fiber (*p* = 0.002, Mann–Whitney *U* test) latencies, 7 days post treatment, compared to recordings from trigeminal neurons in animals treated with saline. The whisker plots show the medians with variability outside the upper and lower quartiles. Dots indicate outliers. (**b**) Examples of traces of post-stimulus recordings with subthreshold (0.1 ms, 11 V) and threshold (0.1 ms, 12 V) electrical stimulations of the periorbital region (assessed as the minimum voltage required to induce evoked action potentials). (**c**) Treatment with BiTox/AA significantly increased the mechanical stimulation threshold required to induce an action potential recorded *in vivo* from trigeminal neurons with Aδ- and C-fiber latencies, 7 days post treatment, compared to the mechanical threshold recorded from trigeminal neurons in animals treated with saline. (**d**) Examples of traces of post-stimulus recordings with subthreshold (0.16, 0.4 g) and threshold (0.6 g) von Frey mechanical stimulation of the periorbital region (assessed as the minimum von Frey force required to induce an action potential when applied on the cell’s receptive field)

The trigeminovascular model of migraine is a useful *in vivo* rodent model that utilizes direct stimulation of trigeminal neurons through stimulation of trigeminal fiber endings innervating meningeal vasculature. This model is particularly useful in the assessment of anti-migraine effects of established and potential treatments [40]. To investigate the actions of BiTox/AA in the trigeminovascular model of migraine, BiTox/AA (20 ng) or saline was injected in a blinded and randomized fashion in the periorbital region of the rats. Seven days later, primary neurons with a biphasic action potential and a receptive field corresponding to the periorbital region were recorded from the trigeminal ganglia. First, the electrical stimulation threshold required to induce an action potential from each neuron was assessed following trigeminovascular activation with increasing voltage [40]. Upon registration of an action potential, the latency was calculated, and cells were classified as Aδ-fiber (*n* = 73 in 14 animals) or C-fiber (*n* = 18 in 13 animals) nociceptors. In this trigeminovascular activation model, both Aδ- and C-fiber nociceptors recorded from BiTox/AA-treated animals displayed a significantly higher electrical stimulation threshold (median for Aδ fibers = 18 V; median for C fibers = 20 V; Fig. 4a, b) compared to neurons recorded from saline-treated animals (median for Aδ fibers = 12; median for C fibers = 14.5 V; Aδ fibers: *p* < 0.001, *r* = − 0. 7; C fibers: *p* = 0.002, *r* = − 0.73) (Fig. 4a, b).

The same neurons were then tested for their mechanical activation thresholds by applying von Frey hair with increasing force on the receptive field found in the periorbital region. A total of 48 Aδ fibers and 17 C fibers displayed an action potential in response to mechanical stimulation. Activation thresholds following mechanical stimulation were significantly higher for both Aδ and C fibers recorded from animals treated with BiTox/AA (median for Aδ fibers = 2.5 g; median for C fibers = 7 g) compared to neurons recorded from saline-treated animals (median for Aδ fibers = 0.07 g; median for C fibers = 1.4 gr; Aδ fibers: *p* < 0.001, *r* = − 0. 72; C fibers: *p* = 0.001, *r* = − 0.81; Fig. 4c, d).

### Effects of BiTox/AA in the GTN Migraine Model

**Fig. 5:**
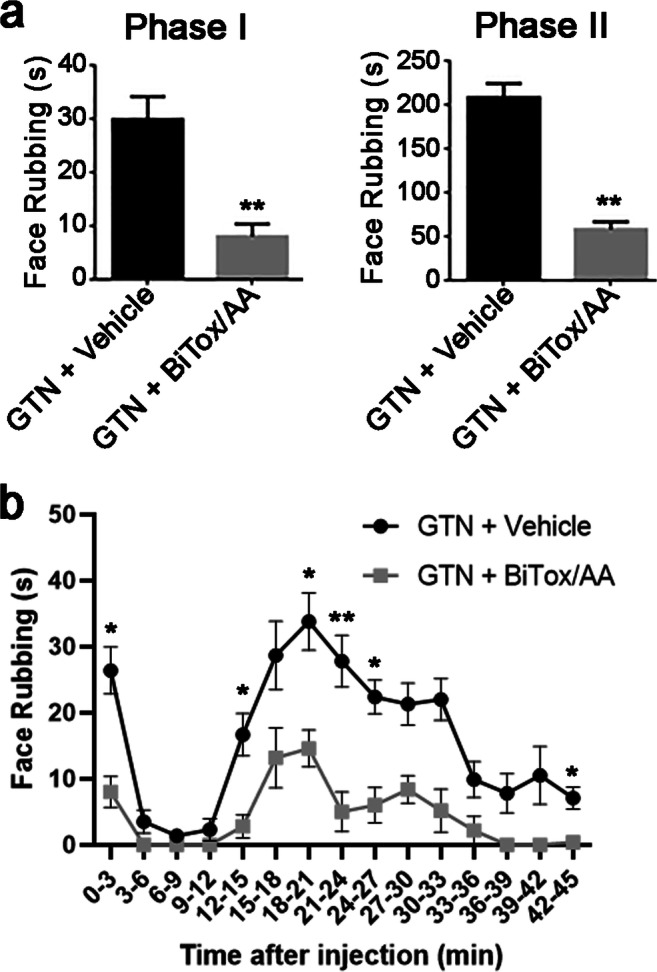
Effects of BiTox/AA in the orofacial formalin test in the glyceryl trinitrate (GTN) animal model of migraine. Bar charts showing the effect of BiTox/AA on total time (in seconds) spent on face rubbing in phases I and II (**a**) and the time course of the face rubbing (**b**). Face rubbing was evaluated by counting seconds animals spent grooming the injected area with the ipsilateral forepaw or hindpaw in the periods 0–6 min (phase I) and 12–45 min (phase II) after formalin injection. The observation time was divided into 15 blocks of 3 min each (45 min total). In both phase I and phase II, pretreatment with BiTox/AA significantly reduced the GTN-induced increase in face rubbing time compared to pretreatment with vehicle (GTN + vehicle) (*p < 0.05, **p < 0.01, unpaired *t* test)

Nitroglycerin, also known as GTN, is a nitric oxide donor that can trigger migraine attacks in patients and is an established model in testing anti-migraine drugs, both in humans and in animals [41]. GTN injection in rodents is known to induce sensitization that can be seen behaviorally as well as upon activation along the trigeminal system [40]. In agreement with previous data [42, 43], in GTN-treated animals, the orofacial formalin injection induced a significant increase in the nocifensive behavior (face rubbing) as compared to rats treated with GTN vehicle (data not shown). Pretreatment with BiTox/AA (10 ng) significantly reduced the duration of GTN-induced nocifensive behavior during phase II when compared to the vehicle group (Fig. 5a, b). Interestingly, BiTox/AA also induced a significant reduction of face rubbing during phase I of the test (Fig. 5a).

## Discussion

Novel nociceptive-specific treatments for migraine with better efficacy and safety profiles compared to current medications are an unmet need for patients. Safe preventive treatments for chronic migraine are highly desirable, since nearly 50% of patients consulting headache clinics have daily or near-daily headaches [44, 45].

The precise molecular events that initiate migraine are not fully understood; however, it is generally agreed that inhibition of the peripheral trigeminal fibers is of pivotal importance in the treatment of migraine. Stimulation of trigeminal fibers in humans produces headache-like pain [46]. A key manifestation of migraine is now considered to be the activation, or the perception of activation, of these fibers [47]. The key pathway for migraine pathophysiology is the trigeminovascular input from trigeminal afferents innervating dural vessels via the trigeminal ganglion to the trigeminocervical complex (TCC), which is the key relay center for the transmission of nociceptive information to higher brain centers where pain sensation is perceived. Notably, sumatriptan and ergotamine do not cross the blood–brain barrier [48, 49], neither do the newer developed monoclonal CGRP antibodies [50]; however, they successfully block the trigeminal system in animal models of migraine and are effective migraine treatments [43, 51, 52].

Here, we engineered and evaluated a novel molecule based on the botulinum neurotoxin. BiTox/AA, a novel elongated BoNT/A prototype molecule with double binding domains, significantly suppressed the frequency of firing of trigeminal fibers in *ex vivo* semi-skull preparations. In the trigeminovascular model of migraine where primary neurons were recorded from the trigeminal ganglia of animals treated with BiTox/AA or saline in the periorbital area, we observed significantly higher activation thresholds upon electrical and mechanical stimulation of both Aδ and C fibers in animals treated with BiTox/AA. At the behavioral level, BiTox/AA reduced the hyperalgesic response to orofacial formalin in GTN-sensitized animal, an established behavioral model of trigeminal hyperalgesia relevant for migraine pain [41]. We observed that BiTox/AA significantly blocked GTN-induced sensitization, as demonstrated by the marked reduction of the nocifensive behavior during phases I and II of rats treated with GTN and subsequently exposed to the orofacial formalin test. Several reports showed that GTN induces spontaneous-like headache attacks in migraine sufferers [53], possibly related to sensitization phenomena, as suggested by neurophysiological investigations in healthy subjects [54]. Peripheral and central sensitizations are thus considered to be important components of the maintenance of migraine [55–57]. Hence, a treatment like BiTox/AA that suppresses trigeminovascular activation and trigeminal nociceptive behavioral in the absence of the muscle paralytic effects represents a promising preventive treatment for migraine. Of note, BiTox/AA had 50 times better efficacy in cleaving SNAP-25 in human neuronal cultures compared to BiTox/A with the single botulinum binding domain [38] (data not shown) which likely allowed for the analgesic effects in low nanogram range seen here. Future work will need to establish the minimal doses of BiTox/AA required for therapeutic effects, maintaining the reduction of migraine in the absence of side effects.

*In vivo* motor control testing revealed that BiTox/AA is 100 times less paralyzing than BoNT/A and does not induce a generalized muscle weakness even at 100 ng dose which would be a fatal dose of native BoNT/A in rats. This could be explained by the increased size of BiTox/AA and thus reduced ability to enter the tight neuromuscular junctions (NMJs) and/or small synaptic vesicles operating in NMJs. The absence of a paralyzing effect for BiTox/AA has a definite clinical relevance when considering that the muscle paralyzing effect of BoNT/A occurs even at very low doses [24]. The possibility to use BiTox/AA at doses comparably higher than BoNT/A may provide additional clinical benefits to migraine sufferers, for example by inducing a response in BoNT/A non-responders or increasing the therapeutic effect in BoNT/A partial responders. Given the expanding knowledge we gained on migraine pathophysiology in recent years, the muscle paralysis induced by BoNT/A is unlikely to contribute towards its efficacy [58], making it an unwanted side effect that potentially limits the efficacy of this treatment. BiTox/AA has reduced toxicity, and hence, it is realistic to hypothesize that a higher therapeutic efficacy could be achieved using higher doses without increased safety concerns for patients. However, a possible immunological response will need to be considered carefully in such future translations.

Our results show that BiTox/AA can cleave SNAP-25 similar to BoNT/A and at a similar rate in human SiMa neuroblastoma cultures (Fig. 2a). BiTox/AA also cleaves SNAP-25 in cultures of rat trigeminal ganglion neurons, including a subset of CGRP-expressing neurons (Fig. 2e). Although what triggers a migraine attack is yet to be understood, the trigeminal system is of pivotal importance in sustaining head pain [47, 59, 60]. CGRP released from the trigeminal system has been implicated in migraine pathophysiology as its levels were found to be elevated during migraine attacks, as well as in patients with chronic migraine [61]. BoNT/A treatment in patients with chronic migraine reduces interictal CGRP plasma levels [62, 63]. Sumatriptan, a well-established migraine treatment, reverses CGRP increase in migraineurs [64], while CGRP receptor antagonists are often effective in treating acute attacks of migraine [65, 66]. BiTox/AA significantly increased the threshold of mechanical and electrical stimulation of both Aδ and C fibers (Fig. 4). Pertinently, BoNT/A was suggested to attenuate the release of CGRP from nociceptors and also to inhibit the release of glutamate [27], the main excitatory neurotransmitter of trigeminal fibers, and a major sensitizing molecule [67]. It will be important to investigate in future experiments how BiTox/AA could affect glutamatergic excitation during nociceptive stimuli which was shown to be exacerbated in the presence of CGRP [68].

Recently, a series of CGRP antagonists and monoclonal antibodies against the CGRP system demonstrated therapeutic benefits in several controlled clinical trials conducted on large populations [43, 69]. However, the widespread expression of CGRP and its receptors throughout the human body [70] could lead to unwanted side effects, including a cardiovascular risk [71–73]. In contrast, BoNT/A action is restricted to nociceptors in peripheral trigeminovascular neurons [29]. A possible distal effect of BoNT/A has been proposed, as a result of its transport to centrally projecting trigeminal terminals where it inhibits transmitter release, thus decreasing the activation of second-order neurons in the trigeminocervical complex [74]. However, this distal action should not raise safety issues if botulinum molecules only affect nociceptive pathways. Together, a localized BoNT/A-based treatment, with a possible blockade of CGRP release [28] from trigeminal fibers near the injection sites and potentially from centrally projected fibers that are restricted to the nociceptive pathways, may represent a more efficient and safer treatment approach than the systemic antibody-based CGRP blockade.

In conclusion, BiTox/AA has a potent SNAP-25 cleavage activity but exhibits a reduced paralytic action. This novel botulinum construct showed significant efficacy in animal models in blocking activation of the trigeminal system and in suppressing trigeminal hyperalgesia. This prototype molecule therefore paves the way for a safe preventive treatment for migraine with enhanced therapeutic effects as compared to currently available options.

## Supplementary Information

ESM 1 (PDF 444 kb)
